# MAKING “NONESSENTIAL” FAMILY/VOLUNTEER CAREGIVING ESSENTIAL IN LONG-TERM CARE HOMES

**DOI:** 10.1093/geroni/igac059.1948

**Published:** 2022-12-20

**Authors:** Annie Robitaille, Linda Garcia, Michaela Adams

**Affiliations:** University of Ottawa, Ottawa, Ontario, Canada; University of Ottawa, Ottawa, Ontario, Canada; University of Ottawa, Ottawa, Ontario, Canada

## Abstract

For individuals living in long-term care (LTC), loneliness is often a concern. With the COVID-19 pandemic, this is only exacerbated as strict restrictions are put in place on visits between residents and their loved ones and on volunteer presence. Understanding how these changes affect residents, family caregivers, and volunteers is paramount to best implement changes with regards to how family/volunteer caregiving presence is managed during pandemics. The objective of this study was to gain a better understanding of the response to COVID-19 that pertains to the family caregiver and volunteer presence in LTC and increase the evidence about the impact of reduced levels of family caregivers and volunteers on residents, caregivers, and volunteers. A total of 64 semi-structured interviews were conducted with caregivers and volunteers. Of these interviews, 49 were one-time interviews and 15 were weekly interviews over a 5-month period to examine the impact of the ever-changing pandemic restrictions on caregivers, volunteers, and residents. Thematic analysis was used to analyze the interviews and two independent researchers coded each interview. Results highlight the importance of connections in LTC, the feeling that human rights were neglected, the importance of flexibility amongst staff, the role of caregivers as advocates for residents, increased caregiver guilt, and resident decline in physical and emotional well-being. The role of family caregivers and volunteers as essential in LTC homes will be discussed and recommendations to revisit policies on the family caregiver and volunteer presence to improve the preparedness for future pandemics and outbreaks will be presented.

